# 1313. Disease Burden and Real-world Clinical Practice for the Treatment of Hospital-Acquired Bacterial Pneumonia Using a Japanese Large-scale Claims Database: A Retrospective Cohort Study

**DOI:** 10.1093/ofid/ofab466.1505

**Published:** 2021-12-04

**Authors:** Masahiro Kimata, Yosuke Aoki, Adachi Noriaki, Takeshi Akiyama, Akiko Harada

**Affiliations:** 1 MSD K.K., Tokyo, Japan, Tokyo, Tokyo, Japan; 2 Saga University, Saga, Saga, Japan; 3 IQVIA Solutions Japan, Tokyo, Tokyo, Japan

## Abstract

**Background:**

With an aging population and increasing healthcare utilization, the frequency of hospital-acquired pneumonia (HAP) is expected to increase. Since HAP is life threatening, appropriate diagnosis and treatment are required; however, large-scale Japanese data focusing on patient profiles and treatment patterns is lacking.

**Methods:**

The demographics and treatment patterns of HAP were examined using a large-scale Japanese claims database from Jan. 2016 to Apr. 2018. The HAP population included patients who received injection antibiotics ≧3 consecutive days after admission, but not within 2 days after admission, and those whose reason for hospitalization was not pneumonia but had a diagnosis of pneumonia after hospitalization (based on ICD-10 codes).

**Results:**

2,968 HAP patients (mean age 77 years, 64.9% male) contributing 2,979 total HAP episodes were included. The 12-month pre-index mean Charlson Comorbidity Index (CCI) score was 4.0±3.1 (mean±SD), CCI scores ≧4 comprised 44.0%. Most HAP episodes (77.6%) occurred ≧5 days after hospitalization. During the 12month pre-index period including outpatients, 84.9% of patients had some type of pneumonia record, 9.1% had VAP (ventilator associated pneumonia) records, and 7.4% had anti-MRSA prescription records. For post-index HAP treatment, ampicillin/sulbactam (36.4%, 8.2±3.8 days) and piperacillin/tazobactam (22.0%, 8.8±4.4 days) were frequently prescribed as the first antibiotic prescription. Ceftriaxone (19.4%) and meropenem (9.8%) were also frequently prescribed. Examinations prescribed during HAP: 30.5% blood culture tests, 28.2% sputum examinations and 29.2% urine antigen tests. The overall mortality rate of HAP in overall hospitalization post-index was 22.0%, in which 14.4% of deaths occurred within 30 days. The mean (±SD) length of overall hospital stay was 49.9 (±34.2) days (11.3 days for HAP period), with 12.4% ICU use and 17.6% ventilator use. The median total cost during hospitalization was ¥1,924,848.18 (&19,248).

**Conclusion:**

The data revealed patient characteristics, treatment patterns, mortality rates and healthcare costs in Japanese HAP patients. This database approach should prove useful for discussing antibiotics usage trends in highly aging Japan.

**Disclosures:**

**Masahiro Kimata, PhD**, **MSD K.K., Tokyo, Japan** (Employee) **Yosuke Aoki, MD, PhD**, **MSD K.K., Tokyo, Japan** (Other Financial or Material Support, Honorarium for Lecturing)**SHIONOGI & Co., Ltd** (Grant/Research Support, Other Financial or Material Support, Honorarium for Lecturing) **Adachi Noriaki, n/a**, **MSD K.K., Tokyo, Japan** (Employee) **Takeshi Akiyama, MSc**, **MSD K.K., Tokyo, Japan** (Independent Contractor) **Akiko Harada, n/a**, **MSD K.K., Tokyo, Japan** (Employee)

